# Prediction of prognosis in T4 or N3 locally advanced nasopharyngeal carcinoma receiving chemoradiotherapy using machine learning methods

**DOI:** 10.3389/fonc.2025.1683501

**Published:** 2025-10-09

**Authors:** Zheng Ma, Weijie Liu, Xiaoya Luo, Xinran Niu, Yanmei Li, Yuanling Ma, Li Hou

**Affiliations:** ^1^ Department of Otolaryngology, General Hospital of Ningxia Medical University, Yinchuan, China; ^2^ Peking University First Hospital Ningxia Women and Children’ Hospital, Yinchuan, China; ^3^ The Second Clinical Medical College, Ningxia Medical University, Yinchuan, China; ^4^ School of Clinical Medicine, Ningxia Medical University, Yinchuan, China

**Keywords:** prediction, nasopharyngeal carcinoma, chemotherapy, radiation, machine learning

## Abstract

**Background:**

This study aims to develop and validate a survival prediction model for T4 or N3 locally advanced nasopharyngeal carcinoma (NPC) patients undergoing chemoradiotherapy (CRT) using machine learning methods.

**Methods:**

A total of 293 patients with locally advanced NPC (T4 or N3 stage) treated with CRT were included in the study. The cohort was divided into a training set (173 patients) and a validation set (120 patients). LASSO regression was used to identify significant prognostic factors, and Cox regression analysis was performed to assess the independent impact of these factors on progression-free survival (PFS). A nomogram was constructed based on the identified prognostic factors to predict 1-, 2-, and 3-year PFS. Model performance was validated using ROC curves, calibration curves, and decision curve analysis (DCA).

**Results:**

The training cohort showed 1-, 2-, and 3-year PFS rates of 92.4%, 81.3%, and 75.2%, respectively. In the validation cohort, the 1-, 2-, and 3-year PFS rates were 90.1%, 83.5%, and 76.0%, respectively, with no significant differences between the groups (P = 0.94). The LASSO-Cox model identified N stage and Epstein-Barr virus (EBV) levels as key prognostic factors. The nomogram demonstrated good discrimination with AUC values of 0.802, 0.709, and 0.686 at 1, 2, and 3 years, respectively. The ROC curve shows the model’s performance with AUC values at 1 year (0.802), 2 years (0.709), and 3 years (0.686), demonstrating the model’s ability to distinguish between different survival outcomes. The calibration curves and DCA confirmed the model’s good agreement with observed outcomes and its clinical net benefit across different risk thresholds.

**Conclusion:**

The survival prediction model based on LASSO and Cox regression provides a robust and interpretable tool for predicting PFS in patients with T4 or N3 locally advanced NPC undergoing CRT.

## Introduction

Nasopharyngeal carcinoma (NPC) is a malignancy that originates in the epithelial cells of the nasopharynx (1). It is notably prevalent in Southeast Asia, particularly in China, with a strong association with Epstein-Barr virus (EBV) infection (2). NPC is often diagnosed at advanced stages, with local invasion and extensive lymph node metastasis being significant features (3). Among the various stages, locally advanced NPC, particularly in T4 and N3 stages, presents a challenge for treatment due to its poor prognosis, despite aggressive therapies such as concurrent chemoradiotherapy (CRT) (4).

Standard treatment for advanced-stage NPC, including T4 and N3, involves CRT, which has improved survival outcomes (5). However, even with this treatment approach, many patients still experience high rates of recurrence and distant metastasis (6). Therefore, accurately predicting survival outcomes for these patients is critical in tailoring treatment strategies to maximize therapeutic benefit and minimize unnecessary toxicity. Traditional prognostic models, which often rely on clinical factors such as tumor size, lymph node involvement, and EBV status, have limitations in predicting individual patient outcomes due to the complexity of disease progression and treatment responses (7–9).

Recent advances in statistical and machine learning methods have provided new avenues for improving survival prediction. Among these, LASSO and Cox regression models have become increasingly popular. LASSO is an effective technique for selecting the most important variables from a large dataset, ensuring the final model is both efficient and interpretable (10, 11). The Cox proportional hazards model, widely used in survival analysis, allows for examining the relationship between various prognostic factors and patient survival outcomes (12).

For locally advanced NPC patients, particularly those with T4 or N3 disease, a survival prediction model based on LASSO and Cox regression can be highly effective (13). By integrating multiple clinical variables, such as age, sex, tumor stage, treatment modalities, and response to therapy, this model can offer a more personalized prediction of patient survival. The LASSO method selects the most significant factors, while Cox regression provides insights into how these factors influence survival outcomes over time (14).

The ability to generate accurate and interpretable survival predictions is essential for clinicians, as it helps them identify high-risk patients early, allowing for the optimization of treatment regimens. By providing more tailored care, this approach has the potential to significantly improve survival rates and quality of life for patients with locally advanced NPC, thereby advancing personalized medicine in this challenging clinical context.

## Method

### Patients

This study retrospectively collected data from 293 patients with locally advanced NPC from three tertiary hospitals in China, covering the period from 2012 to 2020. The inclusion criteria were: 1) a pathological diagnosis of NPC, 2) disease classified as T4 or N3 stage according to the 8th edition of the AJCC staging system, 3) receipt of concurrent chemoradiotherapy (CRT), and 4) availability of follow-up data. Exclusion criteria included: 1) previous treatment with other therapies, such as surgery or non-standard treatments, and 2) incomplete or missing data, which hindered the ability to conduct a comprehensive analysis.

This study was approved by the ethics committee of General Hospital of Ningxia Medical University, and all patients provided informed consent for participation in the study.

### Model construction

Firstly, a LASSO regression analysis was performed to select prognostic factors associated with PFS. Patients were randomly divided into training and validation sets in a 6:4 ratio. In the training set, univariate and multivariate Cox regression analyses were conducted to identify independent prognostic factors associated with progression-free survival (PFS). These independent prognostic factors were then used to construct a nomogram for predicting PFS. The PFS was defined as the time from the initiation of CRT to the first occurrence of disease progression, recurrence, distant metastasis, or death from any cause. Patients without such events at the last follow-up were censored at that time point.

### Model validation

In the validation set, the performance of the model was assessed using receiver operating characteristic (ROC) curves, calibration curves, and decision curve analysis (DCA). In the training set, model performance was further validated using partial dependence plots (PDP), time-dependent variable importance plots, and the Brier score.

### Statistical analysis

Categorical variables and continuous variables were compared using the Chi-square test and appropriate parametric or non-parametric tests, respectively. The risk dependence plot was used to explain the PFS outcomes. Kaplan-Meier (KM) curves were used to analyze the survival rates of the training and validation sets, and Log-rank tests were used to compare differences. All statistical analyses were conducted using R software, and a p-value of <0.05 was considered statistically significant.

## Result

### Baseline

In the total cohort of 293 patients with locally advanced NPC, the distribution of baseline variables is as follows: 77.1% are male, with an average age of 45.6 years, and 53.9% are aged 45 or older. Tumor staging shows that 57.3% of patients are classified as T4, and 49.1% have N3 stage. Regarding EBV DNA levels, 36.9% of patients have levels ≥10000. Upon comparing the training set (173 patients) and the test set (120 patients), no significant differences were observed in the distribution of these variables (**Table 1**).

**Table 1:** Baseline characteristics of the training and validation sets.

Variable	Total (N = 293)	Test (N = 120)	Train (N = 173)	p-value
Sex				**1.000**
Female	67 (22.9%)	27 (22.5%)	40 (23.1%)	
Male	226 (77.1%)	93 (77.5%)	133 (76.9%)	
Age	45.6 (11.1)	45.5 (11.8)	45.7 (10.7)	**0.85**
< 45	135 (46.1%)	61 (50.8%)	74 (42.8%)	**0.214**
≥ 45	158 (53.9%)	59 (49.2%)	99 (57.2%)	
T				**0.376**
T2	29 (9.90%)	13 (10.8%)	16 (9.25%)	
T3	96 (32.8%)	44 (36.7%)	52 (30.1%)	
T4	168 (57.3%)	63 (52.5%)	105 (60.7%)	
N				**0.297**
N1	75 (25.6%)	25 (20.8%)	50 (28.9%)	
N2	74 (25.3%)	32 (26.7%)	42 (24.3%)	
N3	144 (49.1%)	63 (52.5%)	81 (46.8%)	
EBV				**0.930**
<1000	87 (29.7%)	37 (30.8%)	50 (28.9%)	
1000-10000	98 (33.4%)	39 (32.5%)	59 (34.1%)	
≥ 10000	108 (36.9%)	44 (36.7%)	64 (37.0%)	

EBV, Epstein-Barr virus. Bold values P < 0.05 was considered statistically significant.

### Survival

**Figure 1:**
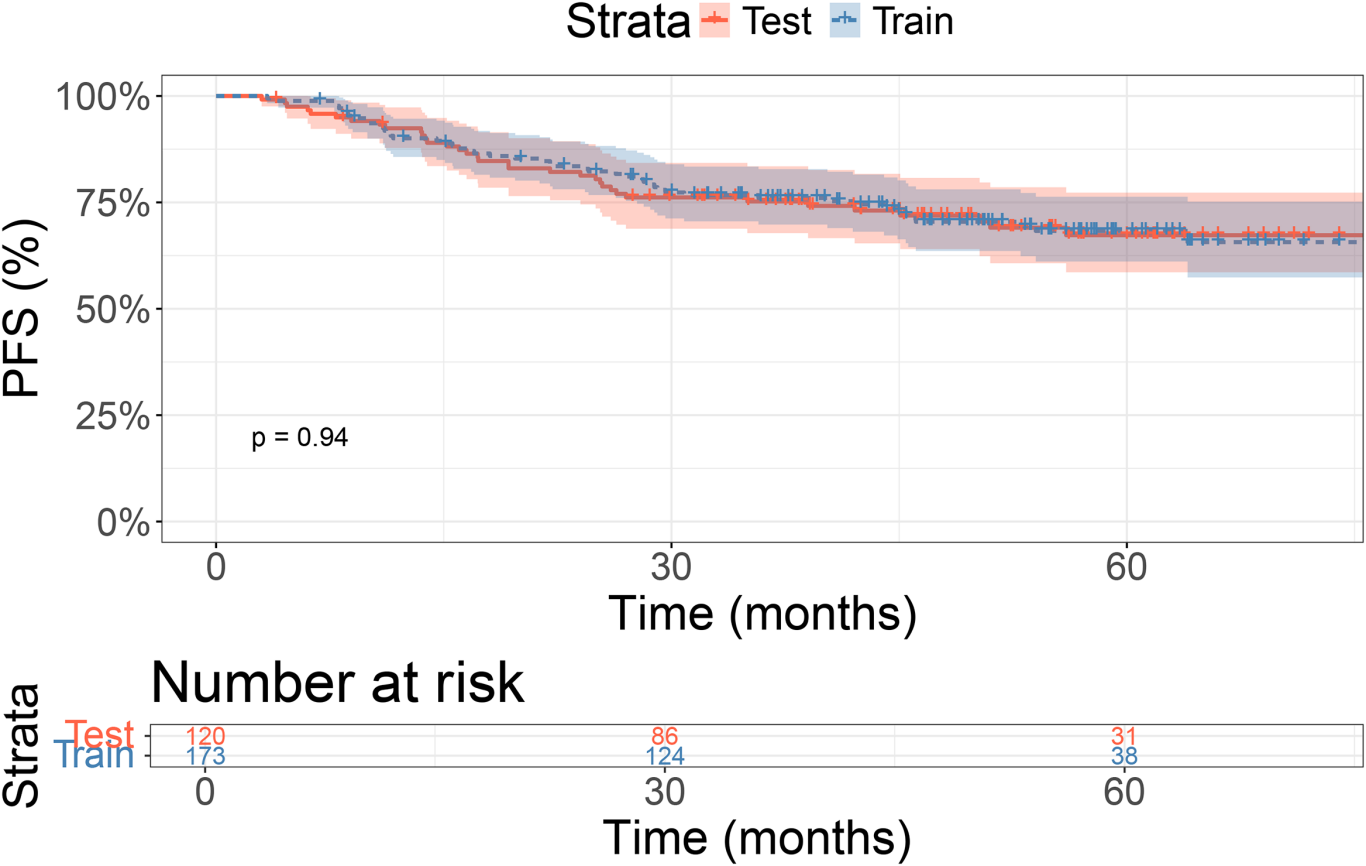
Kaplan-Meier curves for training and validation sets.

In the training cohort, the 1-, 2-, and 3-year PFS rates were 92.4%, 81.3%, and 75.2%, respectively. In the validation cohort, the 1-, 2-, and 3-year PFS rates were 90.1%, 83.5%, and 76.0%, respectively. There were no significant differences in PFS between the two groups (P = 0.94, **Figure 1**).

### Model construction

**Figure 2:**
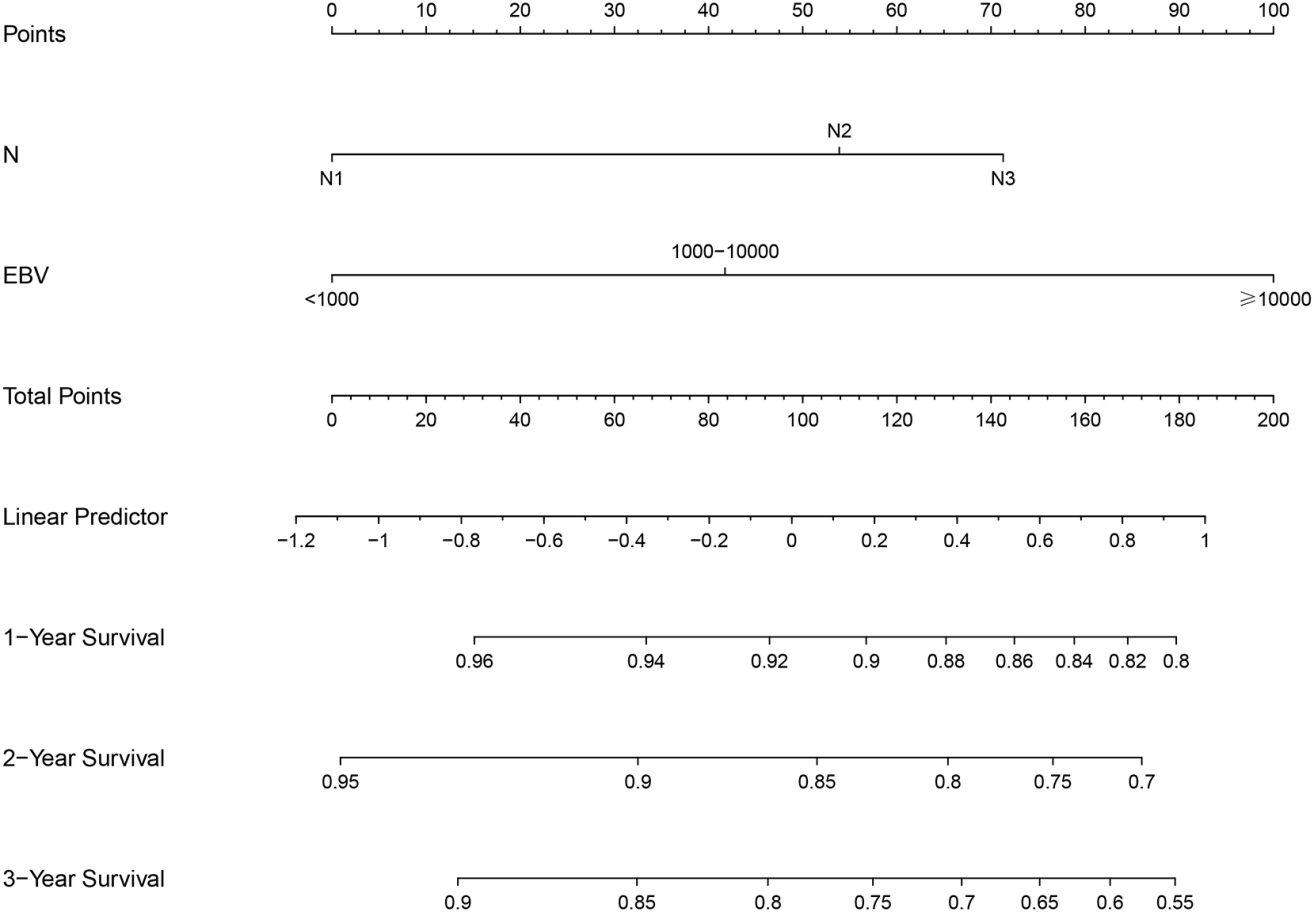
Nomogram construction based on N stage and Epstein-Barr virus levels in the training set.

The LASSO model identified age, T stage, N stage, and EBV as risk factors influencing PFS (**Supplementary Figures 1A, B**). In the training cohort, multivariate Cox analysis confirmed that N stage and EBV levels were independent prognostic factors for PFS (**Table 2**). Based on N and EBV, a nomogram was constructed to predict 1-, 2-, and 3-year PFS (**Figure 2**).

**Table 2:** Univariate and multivariate Cox regression for progression-free survival.

Variable	Category	HR (univariable)	p-value (univariable)	HR (multivariable)	p-value (multivariable)
N	N1	–	–	–	–
	N2	2.01 (0.83-4.85)	0.12	1.85 (0.76-4.50)	0.176
	N3	2.52 (1.15-5.52)	0.021	2.25 (1.02-4.96)	0.044
Age	< 45	–	–	–	–
	≥ 45	1.35 (0.76-2.40)	0.312	–	–
T	T2	–	–	–	–
	T3	1.51 (0.51-4.46)	0.457	–	–
	T4	1.05 (0.37-2.99)	0.931	–	–
EBV	< 1000	–	–	–	–
	1000-10000	1.58 (0.66-3.77)	0.302	1.61 (0.67-3.85)	0.285
	≥ 10000	3.33 (1.51-7.32)	0.003	3.12 (1.42-6.88)	0.005

EBV, Epstein-Barr virus.

### Model validation

The ROC curve shows the model’s performance with AUC values at 1 year (0.802), 2 years (0.709), and 3 years (0.686), indicating varying discrimination ability (**Supplementary Figure 2A**). The calibration curve confirms how well the predicted PFS aligns with observed outcomes (**Supplementary Figure 2B**). The decision curve analysis demonstrates the clinical net benefit of the model at different risk thresholds (**Supplementary Figure 2C**).

### Model interpretation

**Figure 3:**
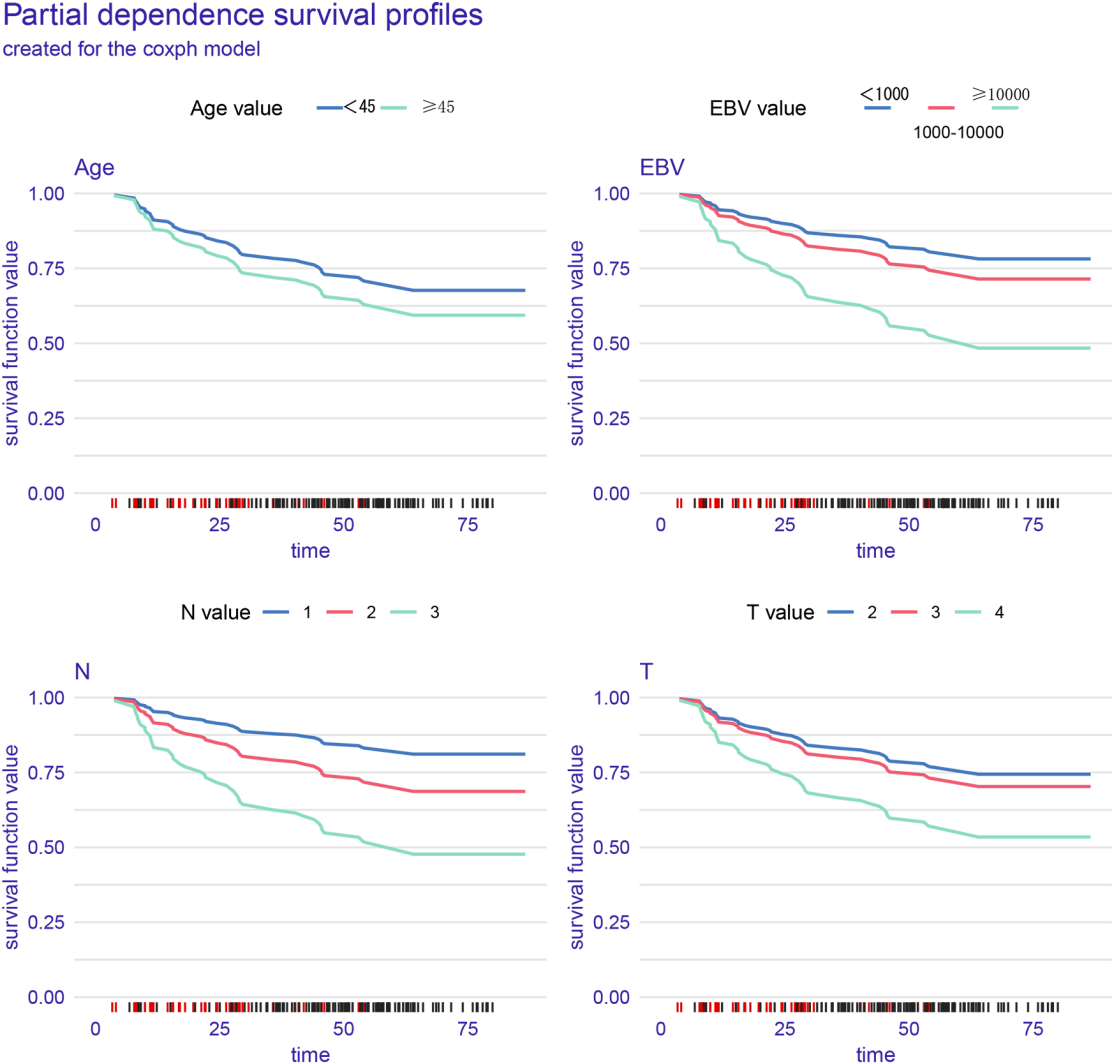
The partial dependence plots (PDPs) in the figure illustrate the relationship between different prognostic factors (Age, Epstein-Barr virus, N stage, and T stage) and progression-free survival (PFS).

**Figure 4:**
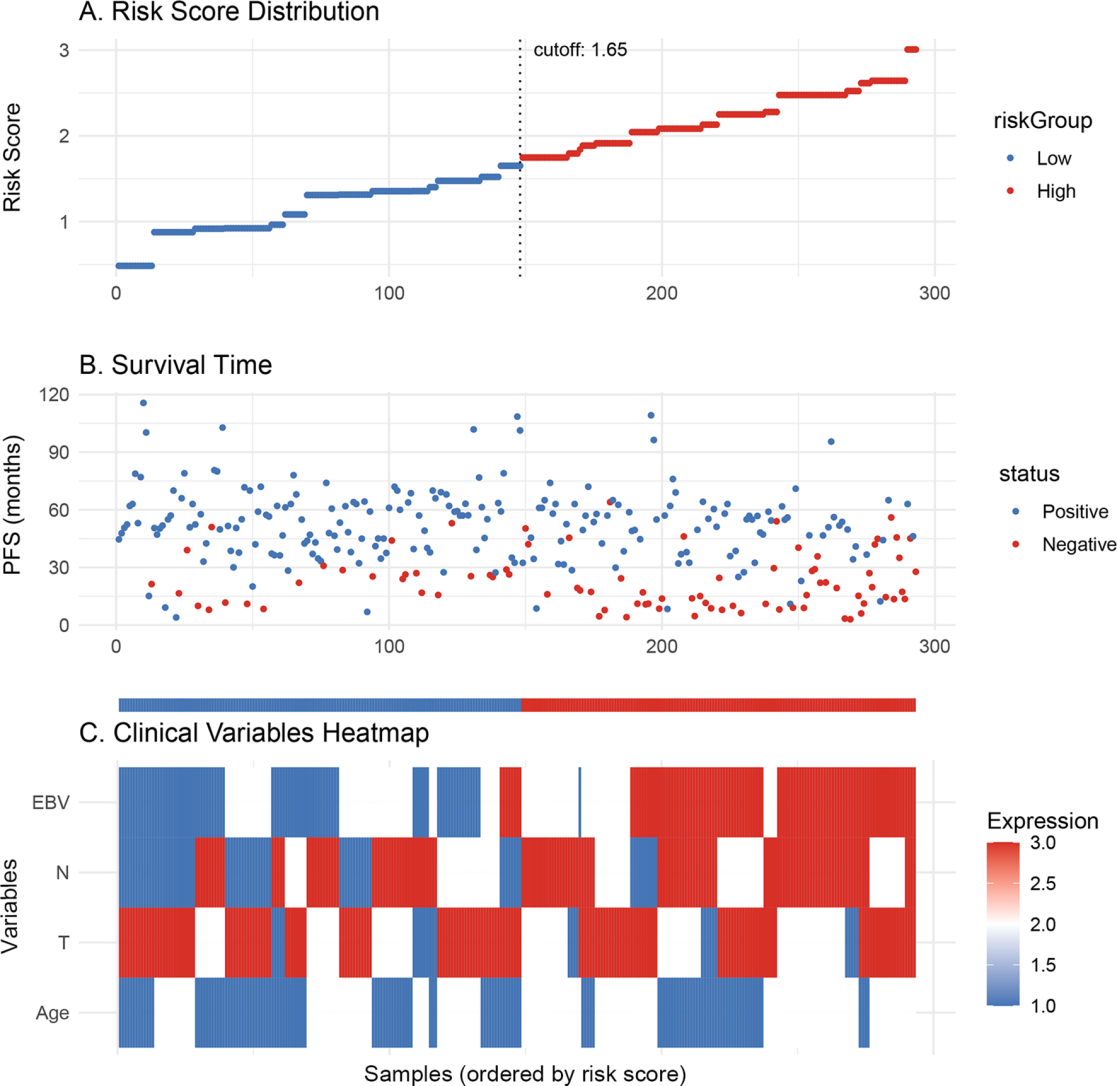
**(A)** Risk scores are divided into low-risk (blue) and high-risk (red) groups based on the cutoff of 1.65. **(B)** Progression-free survival is shown for each patient, with the low-risk group (blue) having longer survival compared to the high-risk group (red). **(C)** The heatmap shows the expression of clinical variables across the risk groups.

In the training cohort, partial dependence plots (PDP) confirmed that higher EBV levels, older age, and more advanced T and N stages were associated with worse survival (**Figure 3**). **Supplementary Figure 3A** shows the model’s performance over time, with the Brier score decreasing, indicating improved prediction accuracy. The graph demonstrates that the model’s ability to discriminate between different survival outcomes improves over time, as indicated by the gradual increase in AUC. **Supplementary Figure 3B** shows that EBV and N stage are the most important factors affecting PFS. **Figure 4A** shows the distribution of risk scores, with a cutoff of 1.65 separating low-risk (blue) and high-risk (red) groups. **Figure 4B** indicates that high-risk patients have shorter PFS, while low-risk patients have longer survival. **Figure 4C** highlights clinical variables (EBV, N/T stage, age), showing higher EBV levels and more advanced stages in the high-risk group.

## Discussion

Locally advanced NPC, particularly those at T4 and N3 stages, presents significant treatment challenges and is associated with poor prognosis (15). Despite the aggressive nature of concurrent CRT, many patients with advanced NPC experience high rates of recurrence and distant metastasis, which worsens their survival outcomes (16). This research is focused on improving prognostication for these high-risk patients by developing a survival prediction model using advanced statistical and machine learning techniques, such as LASSO and Cox regression. By integrating clinical variables, the aim is to enhance personalized treatment planning and offer more accurate survival predictions for patients diagnosed with locally advanced NPC.

The current standard treatment for locally advanced NPC, including T4 and N3 stages, remains concurrent CRT (17). While CRT has shown to improve survival rates, the long-term prognosis for these patients remains suboptimal. High rates of recurrence and distant metastasis suggest that conventional treatment strategies may not be sufficient for all patients, underscoring the importance of developing better prognostic tools to guide treatment decisions (18). Effective prediction models can potentially identify high-risk individuals early, enabling more tailored and aggressive interventions while avoiding unnecessary toxicity in low-risk patients.

In this study, we leveraged LASSO regression to select the most influential prognostic factors, followed by Cox regression for survival analysis. This combination allows for the creation of a robust and interpretable model, which provides both predictive power and clinical applicability. LASSO helps mitigate overfitting by performing variable selection from a broad set of potential predictors, ensuring that only the most relevant factors are included in the final model (19, 20). The use of Cox regression further enhances model interpretability by quantifying the impact of each variable on survival outcomes (21). Additionally, we utilized PDP to visualize the relationship between continuous predictors (e.g., EBV levels and age) and survival, providing valuable insights into how these factors influence prognosis. This feature makes the model more interpretable and clinically relevant, offering a deeper understanding of patient outcomes (22).

The performance of our model is reflected in its ROC curve, with AUC values of 0.802, 0.709, and 0.686 for 1, 2, and 3 years, respectively. This demonstrates good predictive ability and discrimination power, particularly in the short term, which is crucial for clinical decision-making. However, the gradual decline in AUC also highlights the limitations of long-term prediction. Possible explanations include the increasing influence of unmeasured factors (such as genetic or immune characteristics), treatment heterogeneity, and biological variability of the disease over time, all of which may reduce the accuracy of long-term prognostic estimation. Despite these limitations, the model remains clinically valuable: it can help identify high-risk patients with locally advanced NPC who are prone to recurrence or metastasis, thereby guiding clinicians in selecting appropriate treatment regimens and enabling more timely, personalized interventions. Future models incorporating multi-omics or immune-related data may further enhance long-term prediction and improve patient outcomes.

Furthermore, our model may have practical implications in guiding future treatment strategies. Patients identified by the model as having poor predicted outcomes could be considered as candidates for novel therapeutic approaches, such as immunotherapy (23, 24). Recent studies have shown that PD-1/PD-L1 inhibitors provide meaningful clinical benefits in recurrent or metastatic NPC, and ongoing trials are exploring their role in combination with chemoradiotherapy in locally advanced disease (25, 26). By integrating prognostic prediction with treatment selection, our model could help clinicians identify high-risk patients who may benefit from immunotherapy, thereby improving individualized treatment planning.

Despite the promising results, several limitations must be acknowledged (27, 28). Firstly, this study is retrospective, which introduces potential biases inherent in observational studies. The cohort is derived from three tertiary hospitals, which may limit its generalizability to other regions with different patient populations and healthcare settings. Additionally, while we included several clinical variables in the model, the lack of genetic, radiomics, and immune profiling data could reduce the model’s predictive accuracy. Importantly, the study only performed internal validation, and the absence of external validation in independent cohorts limits the robustness and generalizability of the findings. Moreover, detailed information on recurrence sites (local, regional, distant) was not available, which may restrict deeper understanding of prognostic implications. Finally, treatment heterogeneity across hospitals, such as variations in radiation doses or chemotherapy regimens, could influence outcomes and complicate the interpretation of results.

## Conclusion

In conclusion, this study presents a novel survival prediction model for patients with locally advanced NPC, particularly those with T4 and N3 stages.

## Data Availability

The original contributions presented in the study are included in the article/**Supplementary Material**. Further inquiries can be directed to the corresponding author.
