# Supplementary material for: Prediction of prognosis in T4 or N3 locally advanced nasopharyngeal carcinoma receiving chemoradiotherapy using machine learning methods
Source: Front Oncol. 2025 Oct 9;15:1683501. doi: 10.3389/fonc.2025.1683501 (PMC12545415; doi:10.3389/fonc.2025.1683501)

**Supplementary Fig. 1 A.** This plot shows how the model's fit changes with different values of λ. The vertical dashed line marks the optimal λ chosen to minimize model error. **B.** This plot shows how the coefficients of each variable change as λ increases. As λ gets larger, the coefficients shrink, with some variables being reduced to zero.

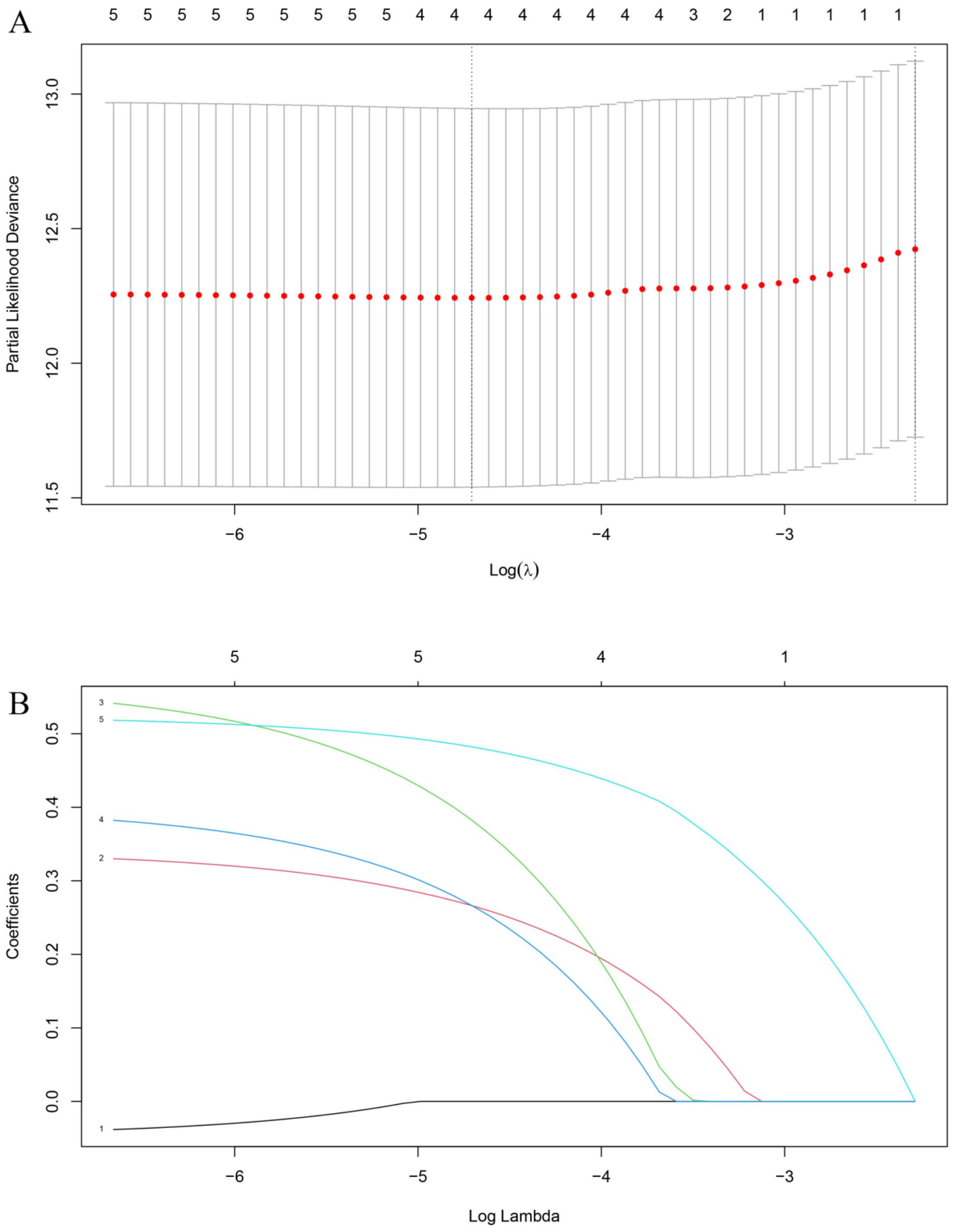

Supplementary Fig. 2 **A.** ROC curves for 1-, 2-, and 3-year PFS with AUC values of 0.802, 0.709, and 0.686, respectively, showing the model's ability to discriminate between survival outcomes over time. **B.** The calibration plot compares the predicted and observed 1-, 2-, and 3-year PFS, with the diagonal line representing perfect agreement. **C. Decision Curve Analysis** shows the net benefit of using the model at different threshold probabilities, indicating its clinical utility in guiding treatment decisions.

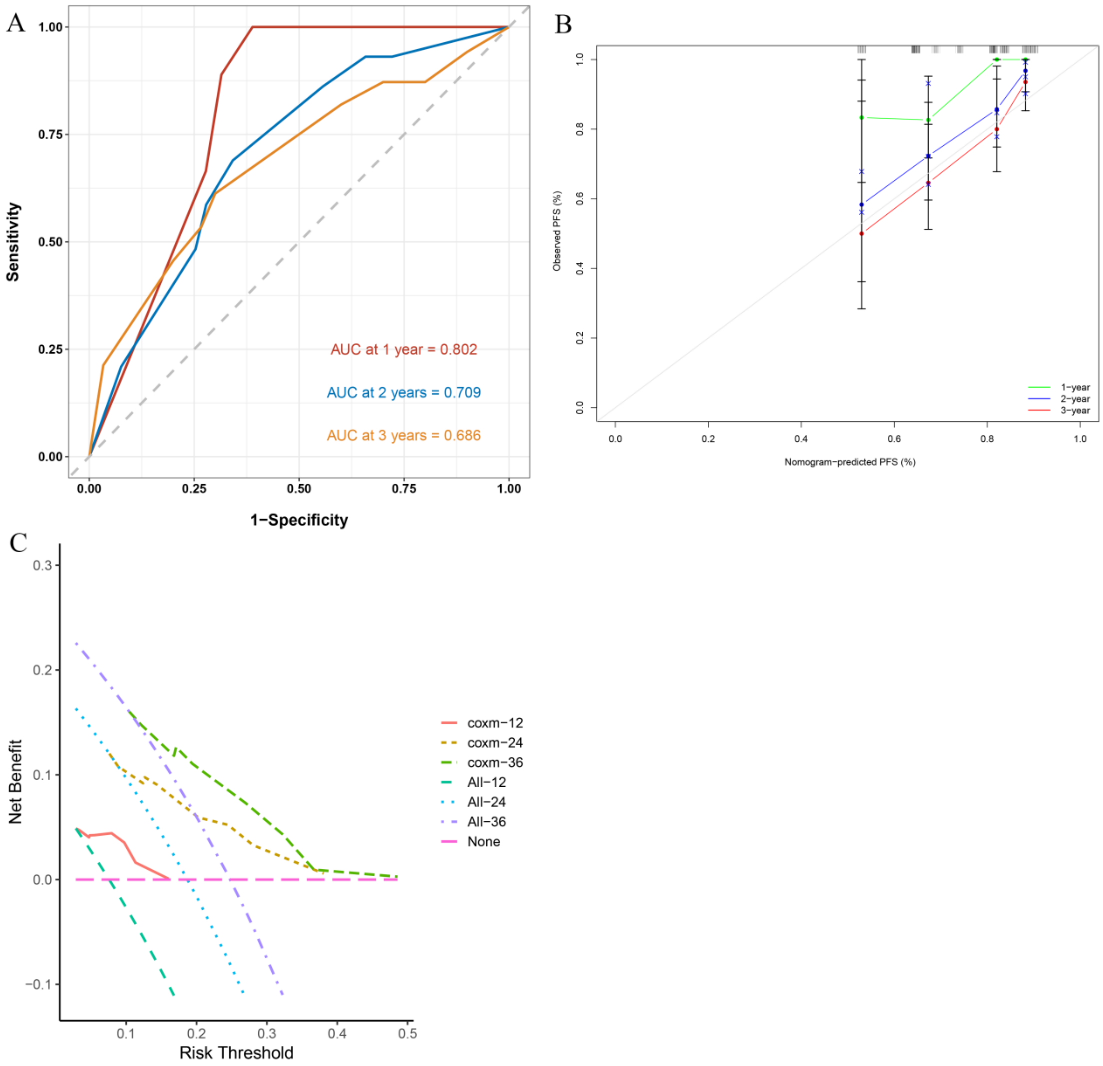

Supplementary Fig. 3

A. Br**ier Score**: This plot shows the Brier score over time, assessing the accuracy of the survival predictions. A lower Brier score indicates better predictive performance.

**Right Panel**: The plot shows the time-dependent C/D (concordance) AUC, demonstrating the model's ability to discriminate between events and non-events at various time points.

**B. Time-Dependent Feature Importance**: This plot displays the time-dependent importance of different features in the survival model. The lines represent the impact of each feature on the model's performance over time, with higher values indicating greater importance.

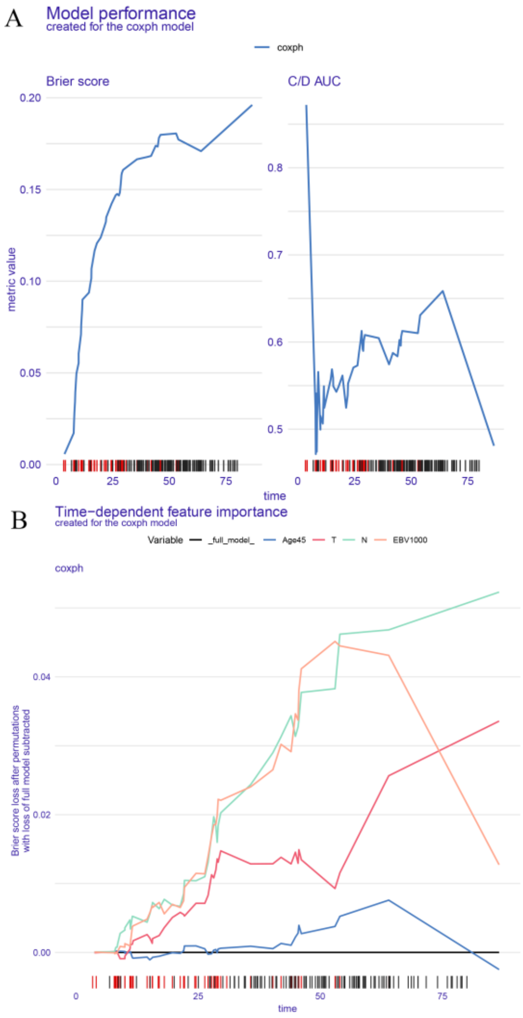

Supplement: Supplementary file 1 [file DataSheet1.pdf]
